# Identification of Expression Patterns and Potential Prognostic Significance of m^5^C-Related Regulators in Head and Neck Squamous Cell Carcinoma

**DOI:** 10.3389/fonc.2021.592107

**Published:** 2021-04-12

**Authors:** Zhenyuan Han, Biao Yang, Yu Wang, Xiuxia Zeng, Zhen Tian

**Affiliations:** ^1^Department of Oral Pathology, Shanghai Ninth People's Hospital, Shanghai Jiao Tong University School of Medicine, Shanghai, China; ^2^National Clinical Research Center for Oral Diseases, Shanghai, China; ^3^Department of Neurosurgery, Huashan Hospital of Fudan University, Shanghai, China; ^4^Department of Stomatology, Putian Hanjiang Hospital, Putian, China

**Keywords:** head and neck squamous cell carcinoma, m^5^C RNA methylation, prognostic signature, TCGA, expression pattern

## Abstract

5-Methylcytosine (m^5^C) methylation is a major epigenetic technique of RNA modification and is dynamically mediated by m^5^C “writers,” “erasers,” and “readers.” m^5^C RNA modification and its regulators are implicated in the onset and development of many tumors, but their roles in head and neck squamous cell carcinoma (HNSCC) have not yet been completely elucidated. In this study, we examined expression patterns of core m^5^C regulators in the publicly available HNSCC cohort *via* bioinformatic methods. The differentially expressed m^5^C regulators could divide the HNSCC cohort into four subgroups with distinct prognostic characteristics. Furthermore, a three-gene expression signature model, comprised of NSUN5, DNMT1, and DNMT3A, was established to identify individuals with a high or low risk of HNSCC. To explore the underlying mechanism in the prognosis of HNSCC, screening of differentially expressed genes, followed by the analysis of functional and pathway enrichment, from individuals with high- or low-risk HNSCC was performed. The results revealed a critical role for m^5^C RNA modification in two aspects of HNSCC: (1) dynamic m^5^C modification contributes to the regulation of HNSCC progression and (2) expression patterns of NSUN5, DNMT1, and DNMT3A help to predict the prognosis of HNSCC.

## Introduction

According to the most recent report, head and neck squamous cell carcinoma (HNSCC) is a relatively lethal type of cancer, and HNSCC ranks among the top six in terms of incidence and mortality, seriously threatening public health and the quality of life of patients with HNSCC worldwide (1). Despite consecutive forms of treatment, namely, surgery, chemotherapy, and radiotherapy, and considerable advancement in the therapeutic schedule for HNSCC, the 5-year survival rate of patients with HNSCC remains far from satisfactory, owing to end-stage diagnosis, rapid development, high recurrence rate, and induction of metastasis to distant sites (2). Mounting evidence has demonstrated that molecular markers hold a promising function not only because of their prognostic value but also because of their role as molecular targets. For example, HNSCC therapy has undergone a significant change in recent years with the development of precision medicines, such as bevacizumab, against vascular endothelial growth factor (VEGF)/VEGF receptor (VEGFR) based on the special gene expression signature, and prognostic outcomes in HNSCC (3–5). More recently, increasing knowledge has brought into focus the features of several macromolecules (protein, RNA, DNA, and sugar) that are involved in tumorigenesis and progression, especially in epigenetic modifications; and the value of these features as prognostic indicators and potential therapeutic targets has been gradually recognized (6–8). To date, the molecular mechanism of HNSCC occurrence and development has not yet been completely elucidated. Therefore, it is imperative to gain a deeper insight into the molecular mechanism of carcinogenesis of HNSCC and thereby provide valuable detection and effective targets for patients with HNSCC.

To date, RNA modification methods have become prominent because of the development of detection technologies and the realization that RNA not only serves as the intermediate molecule for translation or as an auxiliary function for protein synthesis (rRNA and tRNA) but also acts as a functional regulator for the transmission of genetic signals (lncRNA and miRNA) (9, 10). Among these methods, RNA methylation is one of the most common techniques in the epigenetic modification of posttranscriptional RNA, even though extensive effort has been made on studying protein and DNA modifications (11). Generally, RNA methylation predominantly includes m^6^A, m^5^C, m^1^A, and m^7^G, among which m^6^A and m^5^C modification techniques are two of the most major and most representative types of posttranscriptional RNA modification in over 170 chemical modification schemes (12–15). The m^6^A modification technique has been predominantly studied in HNSCC, but that of m^5^C does not attract much attention since the current knowledge of its functions is limited to regulation of the exportation of mRNA and the maintenance of the structure and stability of mRNA (12, 16–18). Its modulating effects have been characterized to be reversible and dynamic, similar to those of histone and DNA modification methods, owing to the involvement of m^5^C writers, erasers, and readers (19, 20). Taking these regulators further, m^5^C was established by adding the methyl group through a number of methyl transferases (NOP2, NSUN2, NSUN3, NSUN4, NSUN5, NSUN6, NSUN7, TRDMT1, DNMT3A, DNMT3B, and DNMT1), was removed by demethylase (TET2 and TET3), and was recognized by binding proteins (ALYREF and YBX1), which were jargonized as “writers,” “erasers,” and “readers,” respectively (12, 13, 21–23).

At present, accumulating evidence has demonstrated that the aberrant expression of m^5^C RNA regulators and specific methylated genes is involved in the pathogenesis of abnormal differentiation in progenitors, fertility damage in males, and cancer oncogenesis (24–27). For instance, DNMT1- and EZH2-mediated epigenetic silencing promotes the progression of glioblastoma and gastric cancer (28). Furthermore, NSUN6 promotes the activation of breast cancer metastasis by incorporating the adaptor proteins, LLGL2, and lncRNA MAYA, to accumulate YAP1 in the nucleus for transcriptional activation (29). In addition, one of the main m^5^C RNA writers, NSUN2, was also noted to be overexpressed in different types of cancer and was deemed an effective prognostic biomarker (25, 27). However, the gene features and prognostic values of m^5^C-related regulators in HNSCC remain obscure and need in-depth investigation.

In this study, we systematically analyzed and evaluated the expression patterns of 15 widely studied m^5^C-related regulators in 501 tumor and 44 normal control tissues and the association between clinicopathological and survival parameters from The Cancer Genome Atlas (TCGA) database.

## Materials and Methods

### HNSCC Dataset Acquisition and Bioinformatic Analysis

The transcriptome TCGA-HNSCC datasets and the corresponding clinical features applied in this study were obtained from the TCGA database *via* the GDC Data Portal, as described earlier (https://portal.gdc.cancer.gov/) (30). In total, 501 tumor and 44 normal control datasets from 527 patients were available for further experimental procedure. In addition, the mutation data and the expression values in the pan-cancer analysis of three selected risk genes were obtained from the cBioPortal database (https://www.cbioportal.org/) and the Gene Expression Profiling Interactive Analysis 2 (GEPIA2) database (http://gepia2.cancer-pku.cn/) (31–33). The differentially expressed genes (DEGs) were generated by the R (3.6.0) package “limma,” the heat map by “pheatmap,” the volcano plots and the bubble plots by “ggplot2,” and the chord plots by “GOplot.”

### Landscape of m^5^C RNA Methylation Regulators

In total, 15 m^5^C-associated regulators composed of 11 writers (NOP2, NSUN2, NUSN3, NSUN4, NSUN5, NSUN6, NSUN7, DNMT1, DNMT3A, DNMT3B, and TRDMT1), two erasers (TET2 and TET3), and two readers (ALYREF and YBX1) were retrieved from the published literature. The expression profile of these 15 regulators, accompanied by clinicopathological parameters, was then systematically extracted and analyzed in patients with HNSCC.

### Consensus Clustering of m^5^C-Related Regulators

To better investigate and construct the distinct classification model, the 13 selected m^5^C-related regulators were screened out to primarily conduct the consensus clustering analysis using the “ConsensusClusterPlus” package of R (3.6.0) (34). Furthermore, the survival analysis of different clusters was performed to determine the best clustering in HNSCC samples.

### Construction of Prognostic Prediction Model

To obtain a better prediction of m^5^C RNA methylation regulators in HNSCC, first, we calculated the hazard ratio (HR), which indicates the result of comparing the hazard function between individuals who are exposed to the hazard function and those who are not, and 95% confidence interval (CI) of the m^5^C-related regulators to identify the appropriate candidate genes by univariate Cox regression analysis. Second, the appropriate candidate m^5^C-related regulators were assigned and built for potential HNSCC prognostic signatures using LASSO Cox regression, which was calculated with the formula described below:

$$\begin{array}{l} RiskScore=\overset{n}{\underset{i=1}{\sum }}coefi\times \;x_{i} \end{array}$$

where *n* represents the number of module RNAs, *coefi* is the coefficient, and *x*_*i*_ denotes the *z-*score-transformed relative expression level (log_2_ (FPKM + 1)) for each gene. Then, the HNSCC cohort from TCGA was divided into two subgroups, high risk and low risk, on account of the median risk score. The Kaplan–Meier survival analysis/risk prediction model was applied to estimate the prognostic values of the candidate risk genes.

### Protein-Protein Interactions and Functional Annotations Analysis

Protein-protein interactions (PPIs) of the 15 m^5^C-related regulators with a combined confidence score > 0.4 and the DEGs between the two risk subgroups were evaluated *via* the STRING database (https://string-db.org/) and visualized using Cytoscape (3.7.1) (35, 36). The Reactome (https://reactome.org/), the Kyoto Encyclopedia of Genes and Genomes (KEGG), and the Annotation, Visualization, and Integrated Discovery (DAVID) databases (https://david.ncifcrf.gov/) were used to evaluate the enriched functional annotations (37–41).

### Immunofluorescence Analysis

Following the guidelines set by the Research Ethics Committee of the Shanghai Ninth People's Hospital, which is affiliated to the Shanghai Jiao Tong University School of Medicine, 10 pairs of tumors, in which the pathological results revealed HNSCC, were selected for this study. Normal human oral mucosal epithelial tissues obtained from the oral mucosa during the surgical resection of HNSCC were used as a control. Paraffin-embedded samples corresponding to the most representative tumor area on H&E-stained slides were selected for performing the IF assay. Briefly, the original fresh-frozen IF sections with a thickness of 6 μm were acquired through cryosection, air-dried for 10 min, fixed with acetone, washed with phosphate buffered saline (PBS) (1 ×), and incubated at room temperature for 2 h with NSUN5 (Proteintech Group, Rosemont, IL, USA, 1:50), DNMT1 (Bioworld Technology, Inc., St. Louis Park, MN, USA, 1:50), and DNMT3A (Novus Biologicals, Littleton, CO, USA, 1:170) primary antibodies. The sections were rinsed with sterile PBS (1 ×) and then incubated with a Goat Anti-Rabbit IgG secondary antibody (Abcam, Cambridge, UK, 1:200) protected from light. Next, the nuclei of the sections were counterstained with DAPI (Beyotime, Shanghai, China). Primary antibodies were replaced with PBS as a negative control.

### Statistical Analysis

Statistical analysis was conducted using R software (3.6.0). Moreover, the DEGs between the two groups were analyzed by the Student's *t*-test. *p* < 0.05 was deemed statistically significant.

## Results

### Expression Patterns of m^5^C RNA Methylation Regulators in HNSCC

**Figure 1:**
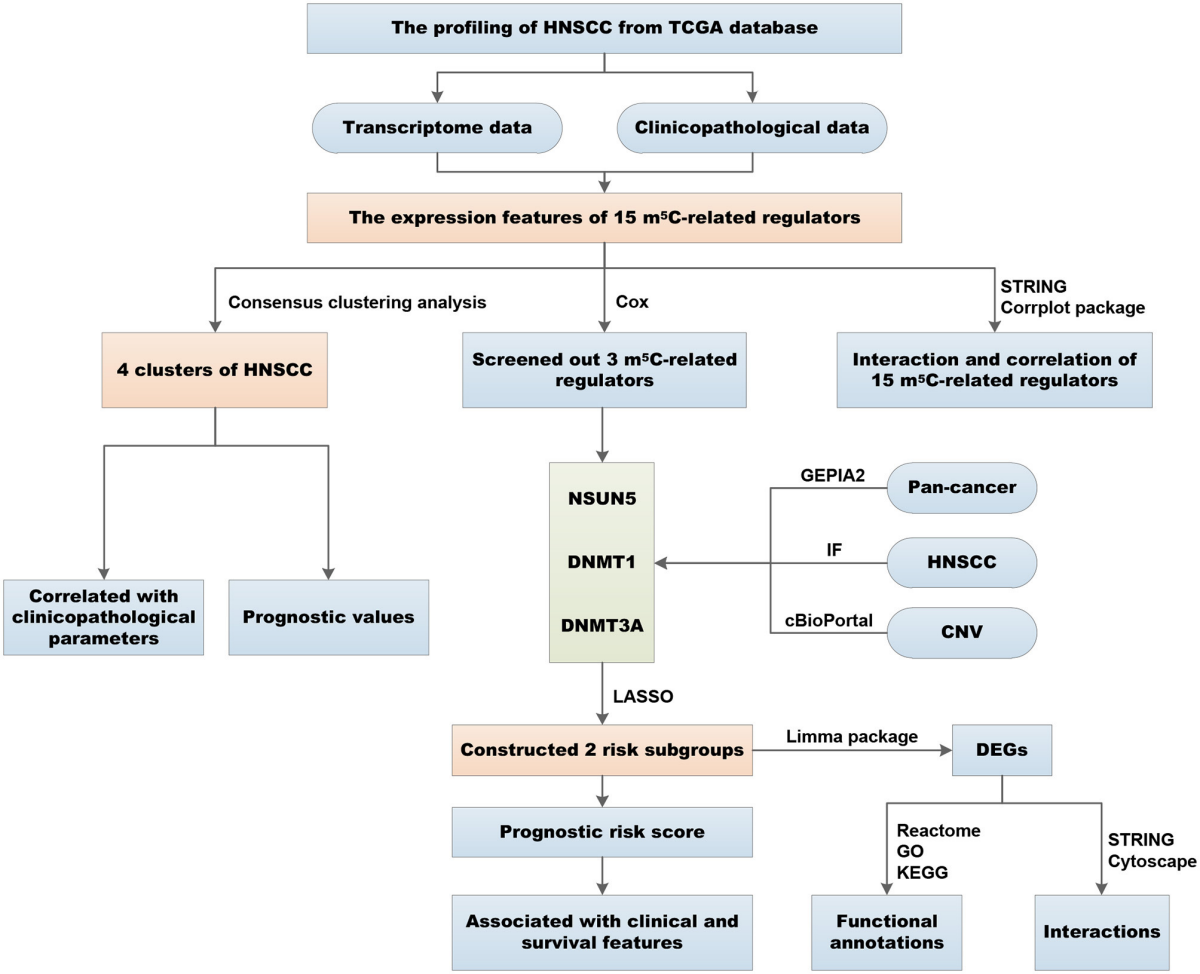
Workflow of the study design and different analyses conducted in the study.

**Figure 2:**
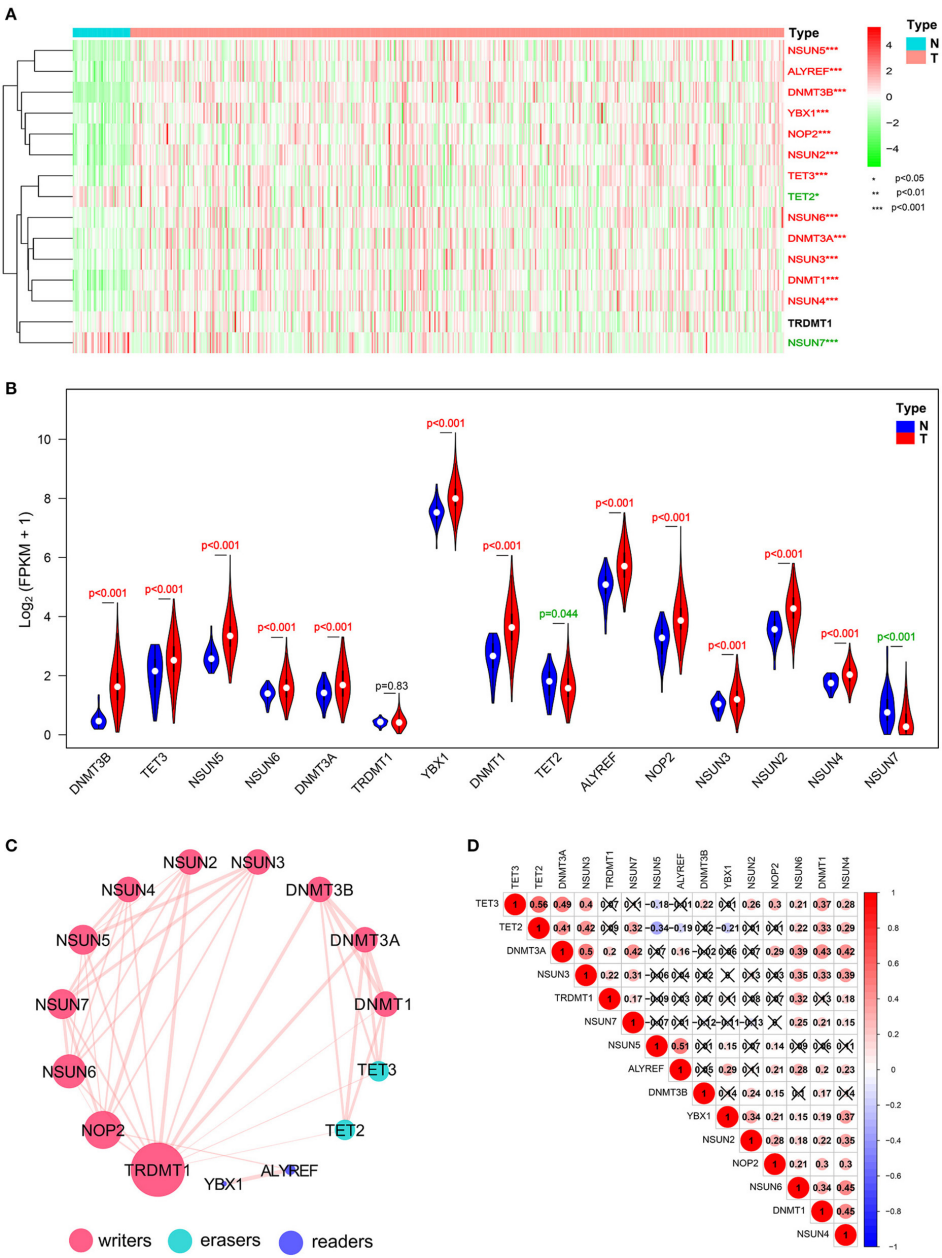
Expressional and interactive landscape of the m^5^C modification signature in head and neck squamous cell carcinoma (HNSCC). **(A)** The heat map shows the expression levels of 15 m^5^C RNA decoration regulators in each clinical sample (N, normal; T, tumor; *p < 0.05; **p < 0.01; ***p < 0.001). **(B)** A violin plot was applied to demonstrate the significantly differentially expressed m^5^C RNA modification regulators between tumor tissues and those of normal control. **(C)** The protein–protein interaction (PPI) network of 15 m^5^C-related regulators was constructed to visualize the interaction (the size of the node is applied to reflect the degree of regulator; the size of the line is applied to denote the combined_score). **(D)** Pearson correlation analysis was delineated to determine the correlation among 15 selected m^5^C-related regulators in The Cancer Genome Atlas (TCGA) HNSCC cohort.

The overall flowchart of the procedure that was applied to in this study and that elucidated the risk score for investigating the prognostic values of HNSCC is summarized in Figure 1. To decipher the expression of essential biological functions of m^5^C-related regulators in HNSCC, we first downloaded and extracted the efficacious gene expression data from the TCGA database. Expression levels of the individual m^5^C RNA methylation catalase in HNSCC and control samples are presented in the heat map (Figure 2A). Among the 545 cases, 14 of the 15 m^5^C methylation regulatory genes were differentially expressed between tumors and healthy samples, with *p* < 0.05. Specifically, NOP2, NSUN2, NSUN3, NSUN4, NSUN5, NSUN6, NSUN7, DNMT1, DNMT3A, DNMT3B, TET2, TET3, ALYREF, and YBX1 exhibited different expression patterns. However, no distinct discrepancies in the TRDMT1 (*p* = 0.83) expression were analyzed in HNSCC tissues when compared with normal tissues (Figure 2B). Of these 14 genes, the majority of writers (NOP2, NSUN2, NSUN3, NSUN4, NSUN5, NSUN6, DNMT3A, DNMT3B, and DNMT1) were more substantially upregulated in the HNSCC samples compared with normal tissues, with the exception of NSUN7, which showed an opposite expression trend with the other 9 m^5^C writers (*p* < 0.05). In addition, in HNSCC tumor tissues, the expression level of the m^5^C eraser TET3 was significantly elevated, while that of TET2 was downregulated. Similarly, the readers ALYREF and YBX1 showed a higher expression level in HNSCC compared to normal tissues. In summary, we concluded that m^5^C RNA methylation regulators had distinct expression changes in HNSCC and corresponding normal tissues.

### Interaction and Correlation Patterns Among the m^5^C RNA Methylation Regulators in HNSCC

To investigate the associations between the main m^5^C RNA methylation regulators, we built a PPI network. The interactions among the 15 m^5^C-related regulators are shown in Figure 2C. TRDMT1 appeared to be the hub gene of the interaction network and was predominantly associated with most of the m^5^C RNA methylation regulators, except for ALYREF and YBX1. Since the PPI network did not provide details of correlation, we performed a further correlation analysis on HNSCC, as shown in Figure 2D. There was a close correlation between TET2 and TET3, two members of the TET gene family. Except for NSUN5 and DNMT3B, NSUN6 was correlated with the other 12 m^5^C RNA methylation regulators. Moreover, the expression of DNMT1 was positively related to the other m^5^C-related methylation genes but not to TRDMT1 and NSUN5. Similarly, NSUN4 also shows a positive relationship with other m^5^C-related methylation regulators except for NSUN5 and DNMT3B. Furthermore, it is worthwhile to note that both erasers, TET2 and TET3, were most negatively correlated with NSUN5 among all the correlations of m^5^C RNA methylation regulators with the Pearson's correlation coefficient of −0.34 and −0.19, respectively.

### Identification of Four Clusters of HNSCC Samples With Different Clinical Outcomes and Characteristics

**Figure 3:**
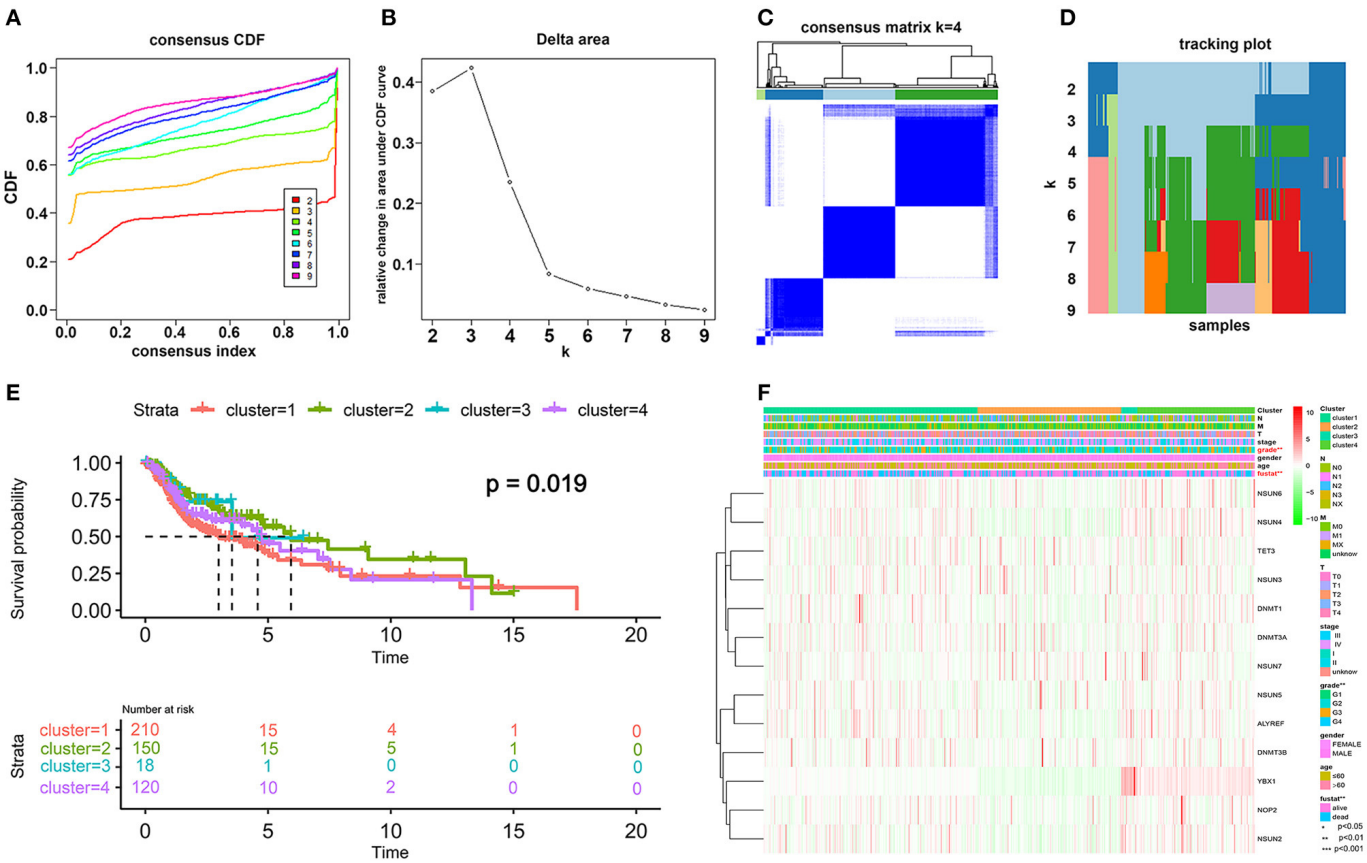
Differential patterns of expressional and clinical features of patients with TCGA HNSCC in cluster 1–4 subgroups. **(A)** Consensus clustering cumulative distribution function (CDF) and **(B)** relative change in the area under the CDF curve from *k* = 2 to *k* = 9. **(C)** Consensus clustering matrix was assessed by *k* = 4. **(D)** The tracking plot of the HNSCC samples. **(E)** The survival analysis for the four clusters by the Kaplan–Meier method. **(F)** The clinicopathological characteristics of the four clusters were determined by coexpression patterns of the consensus expression of 13 selected m^5^C-related regulators (*p < 0.05; **p < 0.01; ***p < 0.001).

Transcriptome data of 545 HNSCC samples from the TCGA database were used for consensus clustering analysis. From the differentially expressed m^5^C-related methylation regulators described above, we used 13 m^5^C-related genes (NOP2, NSUN2, NSUN3, NSUN4, NSUN5, NSUN6, DNMT1, DNMT3A, DNMT3B, TET2, TET3, ALYREF, and YBX1) for further research. Based on the expression similarity profiling of the 13 m^5^C-related regulators combined with consensus clustering cumulative distribution function (CDF) and relative change in the area under the CDF curve, as shown in Figures 3A,B, *k* = 4 was deemed as the appropriate number of clusters when the clustering stability datasets varied from *k* = 2 to *k* = 10 (Figures 3C,D). Then, the HNSCC samples with survival parameters were classified accordingly into four groups. A noticeably shorter overall survival (OS) was observed in HNSCC cases in cluster 1 compared with the other clusters. We highly postulate that the expression of these 13 m^5^C-related genes can divide the HNSCC samples into four groups with distinct prognoses (Figure 3E). Additionally, to better predict the clinicopathological characteristics of HNSCC with these four subgroups, a heat map was applied to present the significant difference in grade and survival condition with both *p* < 0.01, while no huge difference was witnessed with other features, such as TNM classification, gender, age, and clinical stage (Figure 3F). Overall, we can conclude that the expression features of the 13m^5^C-related genes were associated with the grade and survival condition of patients with HNSCC.

### Construction of a Three-Gene Risk Signature With Distinct Prognostic Value

**Figure 4:**
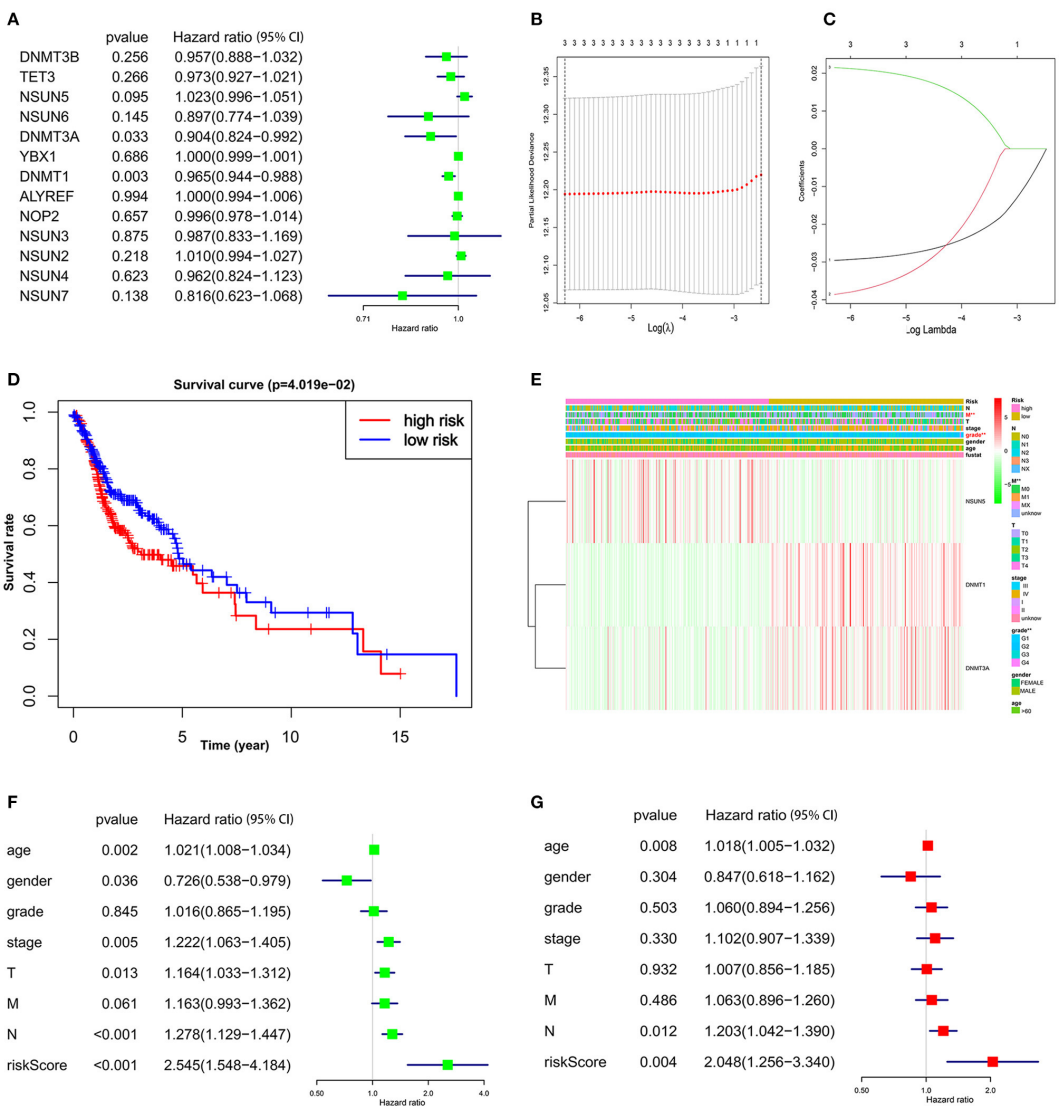
The prognostic risk signature was created with the three m^5^C RNA methylation regulators. **(A)** The process of analyzing the signature regulators by the univariate Cox regression model. **(B,C)** LASSO Cox regression analysis shows the coefficients of the three selected regulators (NSUN5, DNMT1, and DNMT3A). **(D)** The Kaplan–Meier overall survival analysis of the two subgroups of TCGA HNSCC cohorts assigned to high- and low-risk groups followed by risk scores. **(E)** Significant differences were observed in the area of M stage and grade across the high- and low-risk subgroups. **(F)** Univariate and **(G)** multivariate Cox regression showed the relationship between clinicopathological features and the risk score.

To investigate the prognostic role of m^5^C RNA methylation regulators in HNSCC, univariate Cox regression analysis was applied to the 13 m^5^C-related gene expression profiles. According to the details contained in these results (Figure 4A), three (NSUN5, DNMT1, and DNMT3A) of the 13 genes that presented a significant prognostic value (*p* < 0.1) were specifically selected to establish the risk signature. Among these three selected genes, DNMT1 and DNMT3A were protective genes with HR <1, while NSUN5 served as a risk factor with HR > 1.

Then the three screened genes with prognostic values were applied to build the survival risk model using LASSO Cox regression. The coefficients of individual candidate genes were generated based on the minimum criteria (Figure 4B). Subsequently, the risk score of each patient with HNSCC from the TCGA database was calculated as follows: risk score = (−0.029572) *expression of DNMT1 + (−0.038603) *expression of DNMT3A + (0.021496) *expression of NSUN5 (Figure 4C). Afterward, patients with HNSCC were divided into two subgroups, low risk and high risk, according to the median bound. It was observed that patients in the high-risk subgroup had a significantly shorter OS than patients in the low-risk subgroup (Figure 4D, *p* < 0.05). Taken together, these results suggest that the three risk genes can be used as a predictor for the clinical outcomes of HNSCC.

### The Prognostic Patterns of Signature-Based Risk Scores Were Associated With Clinical Features in HNSCC

To evaluate the clinical parameters with the three selected m^5^C methylation regulators in the two subgroups, a heat map was generated to visualize the relationship pattern. The expression of DNMT1 and DNMT3A was high in the low-risk subgroup, whereas, the expression of NSUN5 mainly emerged in the high-risk subgroup. Furthermore, significant differences were disclosed in this heat map concerning the M stage and grade (Figure 4E, *p* < 0.01).

Next, we further performed univariate and multivariate Cox regression analyses to confirm whether the risk signature was an independent prognostic element. Remarkably, age (p = 0.002, HR = 1.021, 95% CI = 1.008–1.034), gender (*p* = 0.036, HR = 0.726, 95% CI = 0.538–0.979), T stage (*p* = 0.013, HR = 1.164, 95% CI = 1.033–1.312), *N* stage (*p* < 0.001, HR = 1.278, 95% CI = 1.129–1.447), and the risk score (*p* < 0.001, HR = 2.545, 95% CI = 1.548–4.184) were determined to be independent prognostic factors from univariate analysis (Figure 4F), while these parameters were calculated by the multivariate Cox regression model. The valuable factors was reduced to age (*p* = 0.008, HR = 1.018, 95% CI = 1.005–1.032), *N* stage (*p* = 0.012, HR = 1.203, 95% CI = 1.042–1.390), and the risk score (*p* = 0.004, HR = 2.048, 95% CI = 1.256–3.340) (Figure 4G).

### External Confirmation of the Expression Patterns and Genetic Alterations of Three Risk Signature Genes in HNSCC

**Figure 5:**
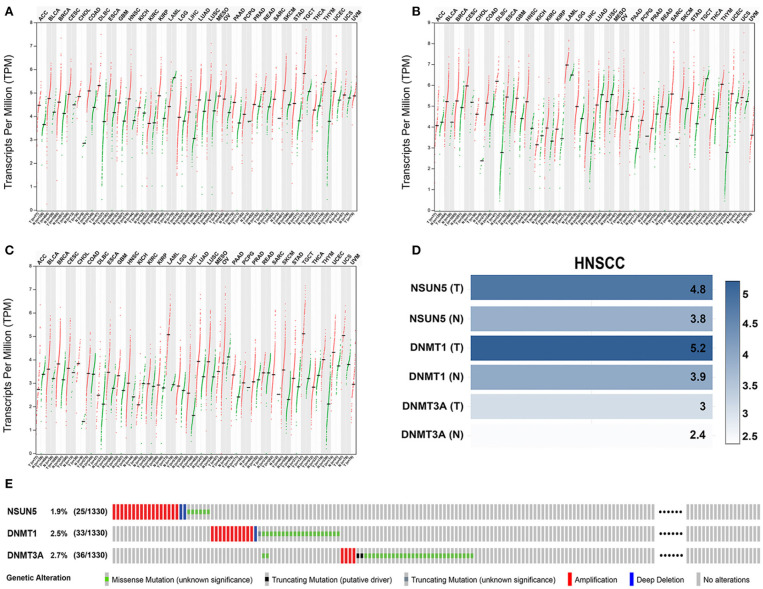
The expression and genetic alteration patterns of three risk genes of various types of cancer in human. The expression values of **(A)** NSUN5, **(B)** DNMT1, and **(C)** DNMT3A in pan-cancers and corresponding normal tissues. **(D)** Expression levels of three risk genes in HNSCC and normal controls based on the GEPIA2 database. **(E)** The profiling of the expression alterations of the three genes in the HNSCC sample (*n* = 1,332) from TCGA datasets.

To better explore the expression profiles of NSUN5, DNMT1, and DNMT3A in human organs and corresponding tumors, the GEPIA2 database was utilized to investigate the distinct organ expression features of these three prognostic markers. As shown in Supplementary Figure 1 and Supplementary Table 1, these three markers were almost overexpressed in tumors compared with normal organs in 26 pairs of comparison. Next, further comparison of the three genes at the transcriptional level among the 31 human pan-cancer samples was conducted, and an almost identical result was obtained from these contrasts. This validation indicates that NSUN5, DNMT1, and DNMT3A were nearly overexpressed in human tumors compared with normal samples (Figures 5A–C).

**Figure 6:**
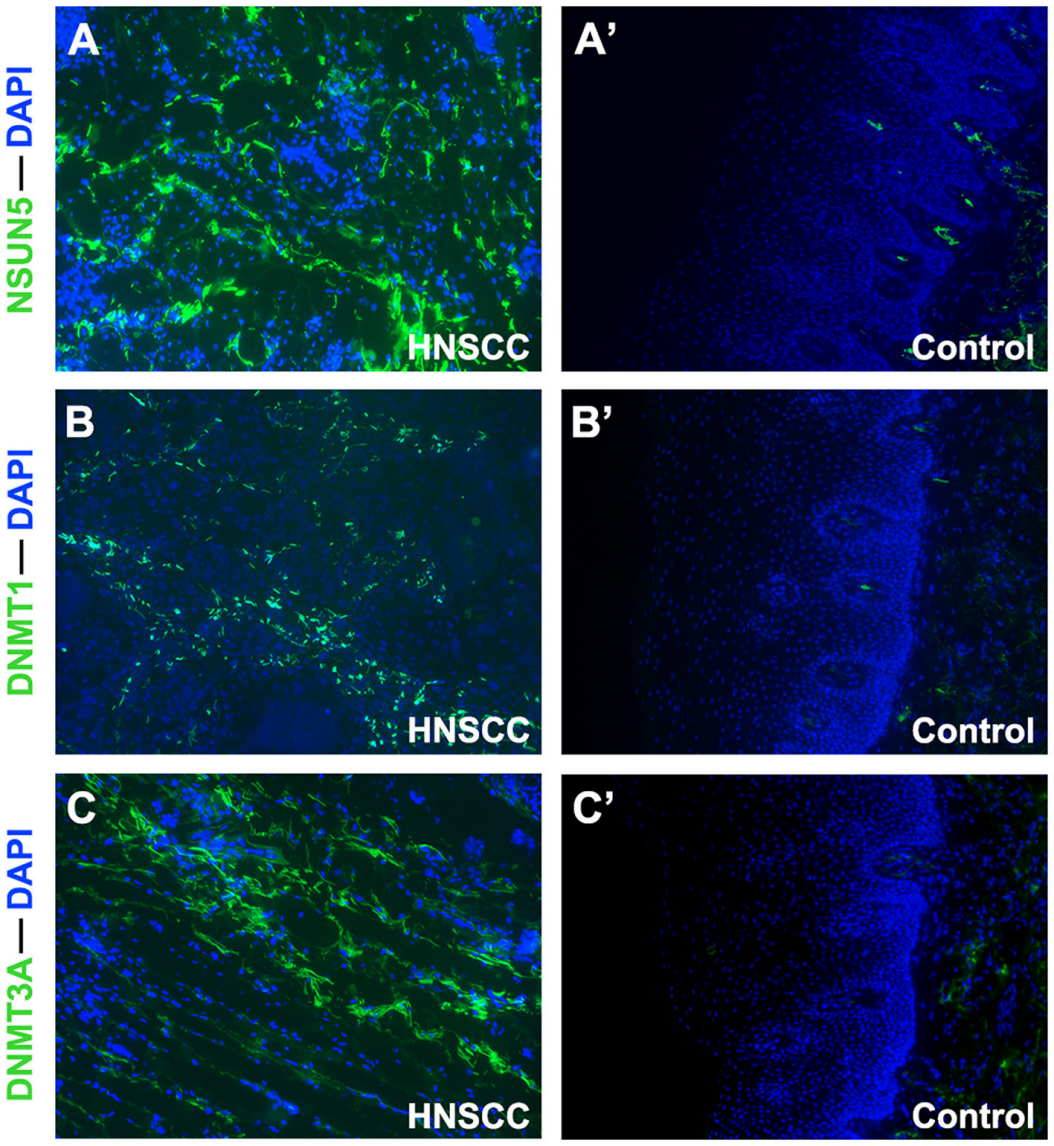
Immunofluorescence analysis of NSUN5, DNMT1, and DNMT3A in tissues of HNSCC tumors and normal oral epithelium (×100). Compared with oral epithelium controls **(A'–C')**, a higher expression of **(A)** NSUN5, **(B)** DNMT1, and **(C)** DNMT3A was detected in HNSCC tumor tissues.

Considering the complexity of the posttranscriptional regulation and the protein expression, we further employed the GEPIA2 database and IF at the protein level to evaluate and verify the expression of three m^5^C signature writers. The GEPIA2 database contains TCGA and Genotype-Tissue Expression (GTEx) data with a larger sample population, which may increase the statistical confidence in a precise estimate. The mRNA expression profiles illustrate that all the three risk signature genes were upregulated in GEPIA2, and similar results were obtained from IF (Figures 5D, 6). Among the 1,330 patients with HNSCC, 92 patients (7.1%) had genetic mutations of NSUN5, DNMT1, or DNMT3A (Figure 5E).

### Functional Annotation and Pathways of Two Risk Subgroups Determined by Three Prognostic Genes

**Figure 7:**
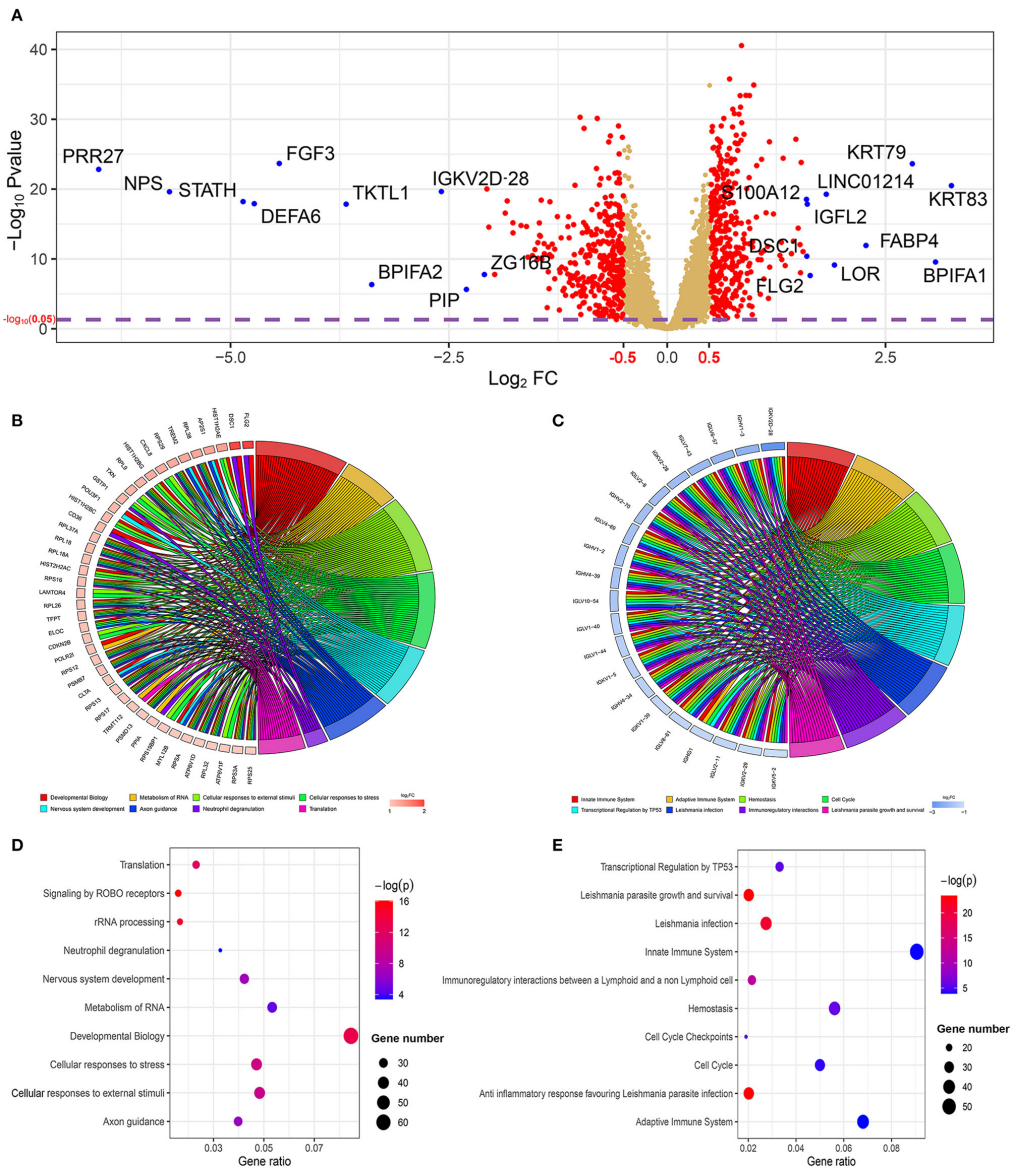
The profiling of differentially expressed genes (DEGs) and their functional annotations from the two risk subgroups. **(A)** The volcano plot shows the DEGs in the two subgroups. The top 10 upregulated and downregulated genes with the highest |log_2_FC| value are labeled with blue dots. Significantly enriched the Reactome pathway analysis of **(B)** 359 upregulated genes and **(C)** 366 downregulated genes was visualized using the package GOplot in R. The plot includes eight Reactome pathway terms. The genes with at least two terms and the terms that included at least eight genes are demonstrated in this plot. log_2_FC represents the difference between the high- and low-risk subgroups. The bubble chart shows the top 10 significant enrichment terms of **(D)** 359 upregulated genes and **(E)** 366 downregulated genes in the Reactome database.

The results mentioned above suggest that the two risk subgroups may be closely related to the prognostic capacity of patients with HNSCC. Next, we sought to explore the potential markers targeting the two subgroups and identified the involved biological functions. In total, 725 DEGs, with 359 being upregulated (*p* < 0.05, log_2_FC > 0.5) and 366 being downregulated (*p* < 0.05, log_2_FC < −0.5), were screened in the high-risk subgroup by the limma package (*p* < 0.05). Among them, we ranked the DEGs followed by log_2_FC. From a total of 20 polar DEGs, 10 upregulated mRNAs (KRT83, BPIFA1, KRT79, FABP4, LOR, LINC01214, FLG2, IGFL2, DSC1, and S100A12) and 10 downregulated mRNAs (PRR27, NPS, STATH, DEFA6, FGF3, TKTL1, BPIFA2, IGKV2D-28, PIP, and ZG16B), were found to be at the top of rankings with the highest |log_2_FC| (Figure 7A). To better understand the interactions between the upregulated and downregulated genes, we also evaluated the PPIs *via* the STRING database and visualized them by Cytoscape (Supplementary Figures 2, 3). In addition, to better summarize the function of DEGs, Reactome, GO, and KEGG analyses were performed to illustrate the functional annotations of DEGs using the GOplot and ggplot2 packages.

**Figure 8:**
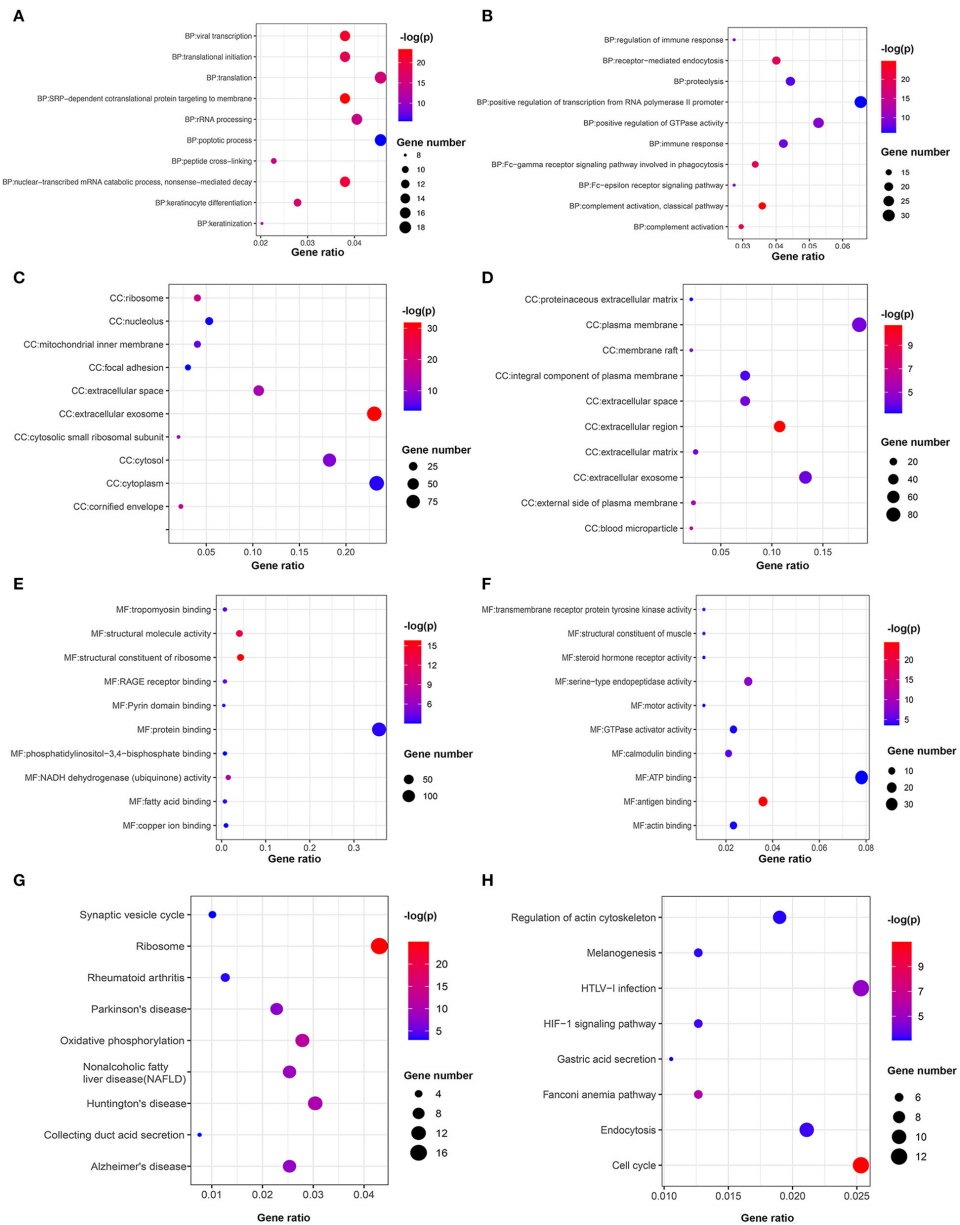
Bubble chart of the enriched terms in GO and KEGG. Functional annotations of upregulated genes in the high-risk subgroup and low-risk subgroup are analyzed by **(A,B)** GO biological processes, **(C,D)** cellular component, **(E,F)** molecular function, and **(G,H)** KEGG pathways.

The GOplot and ggplot2 data of the relationship between the listed 359 upregulated and 366 downregulated genes and their corresponding metabolic pathways in the Reactome database, together with the log_2_FC of the two-part genes, are presented in Figures 7B–E (*p* < 0.05). Furthermore, we also applied ggplot2 to demonstrate the functional annotations in the GO database. The leading highly enriched GO terms of biological process (BP), cellular component (CC), and molecular function (MF) in the high-risk subgroup were “translation,” “cytoplasm,” and “protein binding,” respectively (Figures 8A–E, *p* < 0.05). On the other hand, in the low-risk subgroup, the principal terms of these three aspects degenerated into “positive regulation of transcription from RNA polymerase II promoter,” “plasma membrane,” and “ATP binding” (Figures 8B–F, *p* < 0.05). Moreover, the enriched signaling cascade for the 359 upregulated and 366 downregulated genes identified by the analysis of the KEGG pathway was selected according to log_2_FC (*p* < 0.05). In the high-risk subgroup, the top-ranking terms were associated with “ribosome,” “oxidative phosphorylation,” “non-alcoholic fatty liver disease,” and “synaptic vesicle cycle,” and so on (*p* < 0.05); among them, “oxidative phosphorylation” was testified to be related to larger tumor size of HNSCC (Figure 8G) (42). Verification of the low-risk subgroup with KEGG analysis also revealed that “cell cycle” and “HTLV-infection” were linked with these genes (Figure 8H).

## Discussion

Recently, increasing evidence has demonstrated that HNSCC is a complex and heterogeneous disease that is attributed to the combination of virus infection, environmental risk factors, and genetic predisposition. Of note, tobacco smoking and alcohol abuse are considered to be the leading carcinogenic factors for HNSCC (43). In addition, m^5^C RNA modification in HNSCC has garnered substantial attention among researchers worldwide. Its functions should include numerous BPs, such as mRNA export, RNA stability, translation, and alternative splicing (12, 17, 18, 44–47). m^5^C RNA modification can be detected in most types of RNA and is associated with a wide range of disorders (13, 48). In particular, abnormal m^5^C methylation has been implicated in the development of many malignant tumors, namely human skin squamous cell carcinomas and breast cancer (8). Although, Xue et al. (49) reported that the gene significance of m^5^C regulators can predict the prognosis of patients with HNSCC, the role of m^5^C modification in HNSCC is still obscure, and in-depth investigations in the field are urgent. In this study, we analyzed the expression patterns of 15 m^5^C regulators in HNSCC and constructed a three-gene risk signature to predict the prognosis of patients with HNSCC.

Because of the advancements in sensitive, quantitative, and specific technologies, the identification of modification techniques on the low abundance RNA m^5^C regulators has been brought into focus. Mounting evidence has shown that these regulators, namely m^5^C “writers,” “erasers,” and “readers,” modulate the occurrence and progression of tumors primarily through their methylation function. For example, the well-identified “writer” NSUN5 exhibits tumor-suppressing characteristics in gliomas. DNA methylation-associated epigenetic silencing of NSUN5 is observed in human gliomas, and it helps glioma cells overcome hostile stress conditions (50). Chen et al. (21) revealed that YBX1 is an m^5^C “reader” that recognizes m^5^C-modified mRNAs in human urothelial carcinoma of the bladder (UCB) and maintains the stability of its target mRNAs. Moreover, YBX1 targets the m^5^C methylation site in the HDGF 3′ untranslated region to drive UCB pathogenesis (21). Notably, most m^5^C regulators have been reported to participate in cancer pathogenesis *via* non-methylated pathways. Xu et al. revealed the critical role of YBX1 in modulating abnormal ubiquitination in hepatocellular carcinoma (HCC). Protection of YBX1 from PRP19-mediated ubiquitination degradation by circRNA-SORE, a newly discovered circRNA highly expressed in HCC, increases sorafenib resistance in patients with HCC (51).

In this study, we intend to investigate the expression profile of 15 m^5^C-related regulators in HNSCC. Analysis of the TCGA HNSCC cohort revealed that 14, namely, NOP2, NSUN2, NSUN3, NSUN4, NSUN5, NSUN6, NSUN7, DNMT1, DNMT3A, DNMT3B, TET2, TET3, ALYREF, and YBX1, out of 15 m^5^C-related RNA regulators exhibited different expression hallmarks among tumors and normal controls. Considering that m^5^C regulators are differentially expressed in other tumors and involved in the regulation of their pathogenesis, interpretation of the analyzed results somewhat indicates that the differentially expressed regulators may affect HNSCC development and therapy. For example, the levels of DNMT3B, NSUN2, DNMT3A, NOP2, DNMT1, NSUN4, NSUN5, and ALYREF were upregulated in lung adenocarcinoma (52). Zhang et al. (53) have found that DNMT1 could enhance the radiosensitivity of HPV-positive HNSCC through suppressor of morphogenesis in genitalia 1 (SMG1). The NSUN family member, NSUN2, is found to be implicated in regulating cell cycles and accumulates in a variety of tumor lesions compared with normal samples (25, 54). Therefore, it seems valuable to further investigate the role of m^5^C regulators in HNSCC.

This study attempted to uncover the prognostic effects of m^5^C RNA methylation regulators in HNSCC. We identified four subgroups of HNSCC based on m^5^C RNA methylation regulators by means of consensus clustering and found that the classification was related to OS and tumor grade, implying that the expression pattern of m^5^C-related genes was positively correlated with the malignant process and prognosis of HNSCC. A previous study divided the TCGA HNSCC cohort into two subgroups depending on the 13 m^6^A RNA methylation regulators and applied consensus clustering (55). The OS and tumor grade of patients were also found to be strongly different between two identified subgroups. Taken together, these results indicate that RNA methylation regulators (m^6^A, m^5^C) might be associated with the prognosis of HNSCC.

Afterward, the prognostic three-gene risk signature, comprised of NSUN5, DNMT1, and DNMT3A, was built to effectively distinguish between patients with high risk and patients with low risk and robustly predict OS in the subgroups of HNSCC. Specifically, we observed that low NSUN5, together with high DNMT1 and DNMT3A levels, were positively associated with favorable functional outcomes in patients with HNSCC. Besides, both univariate and multivariate Cox analyses revealed that the risk score could act as an independent prognostic factor in HNSCC, implying that NSUN5, DNMT1, and DNMT3A could be involved in tumor oncogenes and suppressors. It is noteworthy that all three risk genes were m^6^A “writers,” though they showed the opposite effect on survival in HNSCC, which may hint that the NSUN and DNMT family proteins affect the OS in HNSCC with diverse impacts.

Currently, the roles of NSUN5, DNMT1, and DNMT3A in tumors have been widely explored. NSUN5 was reported to be responsible for modifying the second m^5^C position in eukaryotic rRNA and maintaining global protein synthesis and normal growth in mice (56, 57). Another study demonstrated that NSUN5 epigenetic inactivation is a hallmark of long-term survival for patients with glioma (50). Additionally, emerging research has indicated that DNMT1 and DNMT3A share oncogene affections (58, 59). These results agree well with the constructed risk model using the three risk genes. Taken together, the risk model offers a basis for further studies of pathogenesis, and for the determination of the novel classification and construction of the prognosis model of HNSCC.

**Figure 9:**
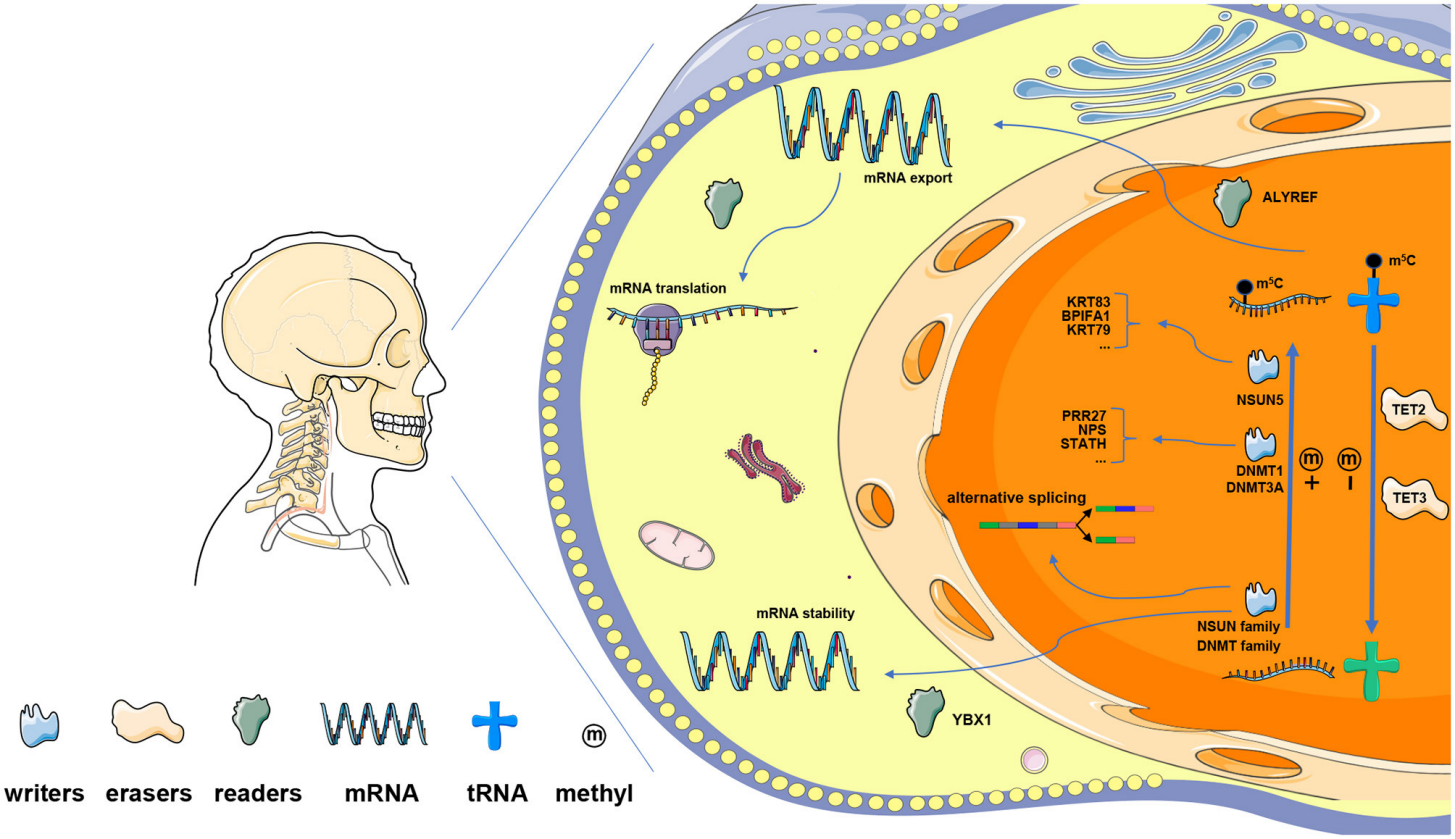
The presumptive outline of m^5^C RNA modification in HNSCC. Methyltransferases of the NSUN family and DNMT family are the main m^5^C “writers.” Among them, NSUN5 is predicted to methylate KRT83, KRT79, and BPIFA1; whereas DNMT1 and DNMT3A target PRR27, NPS, and STATH. TET2 and TET3 serve as m^5^C “erasers,” which regulate RNA m^5^C dynamics together with “writers” in the nucleus. m^5^C readers bind m^5^C-modified RNA and modulate the processes of mRNA translation, alternative splicing, and RNA decay. Specifically, ALYREF functions in the nucleus and regulates mRNA export, while YBX1 maintains mRNA stability in the cytoplasm.

Moreover, the m^5^C-related risk model was found to be associated with the signaling pathways and biological functions of HNSCC. The role of m^5^C RNA regulators was discovered not so long ago. Thus, we identified several functional annotations and signaling pathways related to the two risk subgroups. In the high-risk subgroup, the translation, protein binding, and oxidative phosphorylation terms were enriched by the Reactome, the GO, and the KEGG databases, which were ascertained to be correlated with tumorigenesis (60–62). On the other hand, the top 20 DEGs identified in the two risk groups are probably associated with a high likelihood of underregulation by the three m^5^C writers (NSUN5, DNMT1, and DNMT3A). In detail, the results suggest an m^5^C-regulated mechanism in HNSCC, by which NSUN5 might target KRT83, KRT79, and BPIFA1, while DNMT1 and DNMT3A methylate PRR27, NPS, and STATH. Consistent with these m^5^C “writers,” m^5^C “readers” ALYREF and YBX1 might be implicated in HNSCC by functioning as mediators of the mRNA output from the nucleus and by maintaining the stability of their target mRNAs (12, 21). Based on this, the hypotheses on the role of m^5^C in HNSCC initiation and progression through its internal interactions and signaling pathways have been proposed (Figure 9). Besides, the IF analysis revealed that NSUN5, DNMT1, and DNMT3A were similarly overexpressed at the protein level, which was concordant with the transcriptomic level. This is the first characteristic for detecting RNA methylation regulators using IF. These findings, if verified in a larger cohort of prospective clinical cases combined with prognostic data, might be precise for the forecasting and management of patients with HNSCC. In addition to this shortcoming, we also need to acknowledge another limitation that we came across. Whether m^5^C decoration on RNA was associated with HNSCC prognosis was not directly proven.

## Conclusion

In summary, we systematically illustrated the expression profile, biological function, and clinical prognostic value of m^5^C regulators in HNSCC. The association between m^5^C-related genes and HNSCC progression has been identified. Furthermore, a 3-m^5^C-related gene-based risk score model was built using NSUN5, DNMT1, and DNMT3A, hinting at a prognostic value in HNSCC.

## Data Availability Statement

The publicly available datasets were analyzed in this study. All of the raw data can be found in The Cancer Genome Atlas (TCGA), the Gene Expression Profiling Interactive Analysis 2 (GEPIA2), and the cBioPortal databases.

## Ethics Statement

The studies involving human participants were reviewed and approved by the Ethics Committee of Shanghai Ninth People's Hospital affiliated to Shanghai Jiao Tong University, School of Medicine. The patients/participants provided their written informed consent to participate in this study.

## Author Contributions

ZT conceived and developed the outline of the study. ZH and BY downloaded the data and performed the statistical study. ZH and XZ generated figures and tables and wrote the manuscript. YW revised the paper. All of the authors read and approved the final manuscript.

## Conflict of Interest

The authors declare that the research was conducted in the absence of any commercial or financial relationships that could be construed as a potential conflict of interest.
