# Supplementary material for: Identification of Expression Patterns and Potential Prognostic Significance of m5C-Related Regulators in Head and Neck Squamous Cell Carcinoma
Source: Front Oncol. 2021 Apr 12;11:592107. doi: 10.3389/fonc.2021.592107 (PMC8072008; doi:10.3389/fonc.2021.592107)
Supplement: Supplementary file 1 [file Data_Sheet_1.PDF]

Supplementary Material

Supplementary Figures

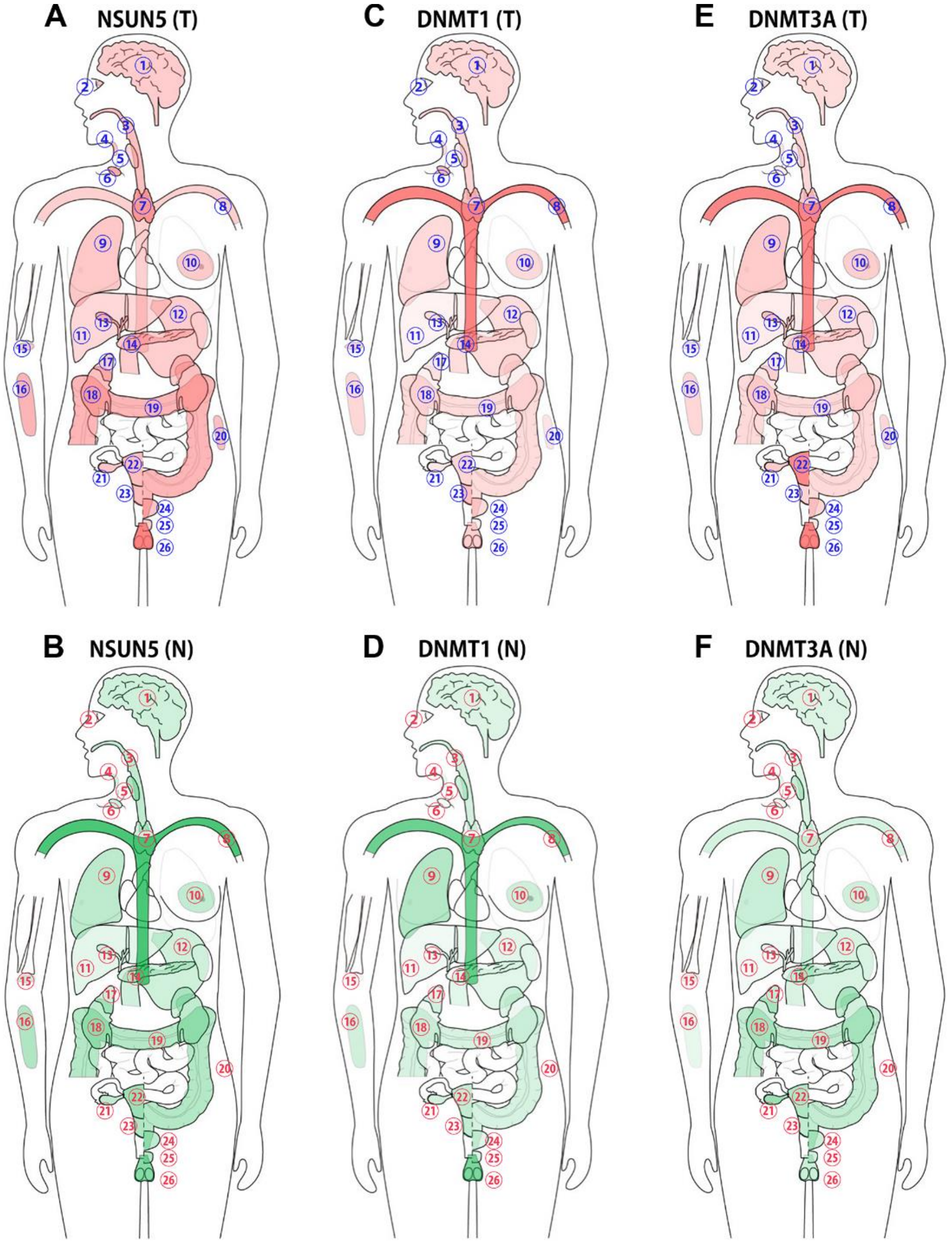

Supplementary Figure 1. The anatomical map showed the expression levels of 3 risk genes in the 26 normal organs and the corresponding tumors. The darker the color, the higher the content of the 3 regulators. The expression values were attached in Supplementary Table 1.

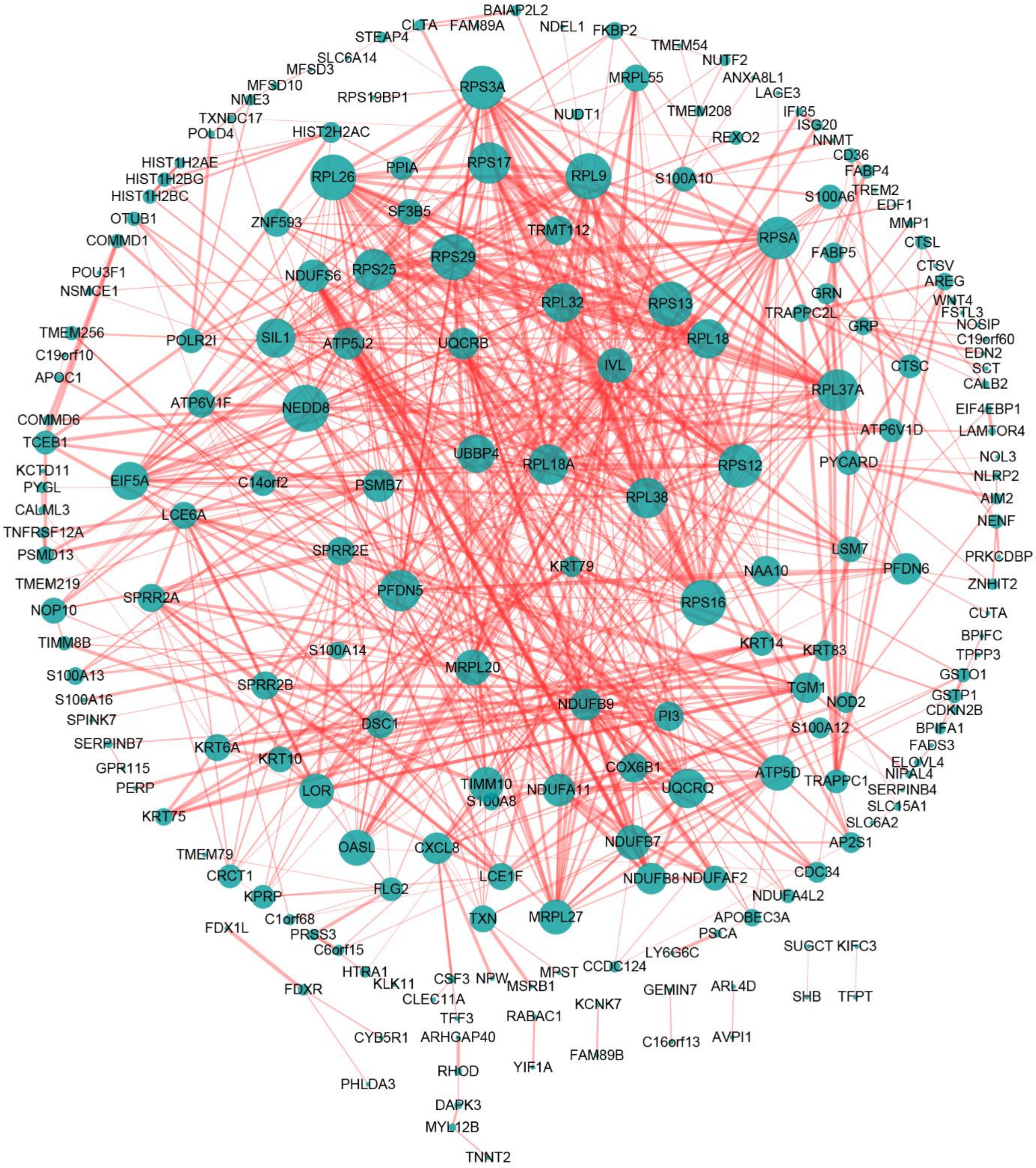

Supplementary Figure 2. The PPI network of high-risk subgroups of up-regulated DEGs genes created by Cytoscape. The node was represented as gene and the edge was deemed as the interactions. The size of the node and edge can be denoted as the degree and combined_score, respectively.

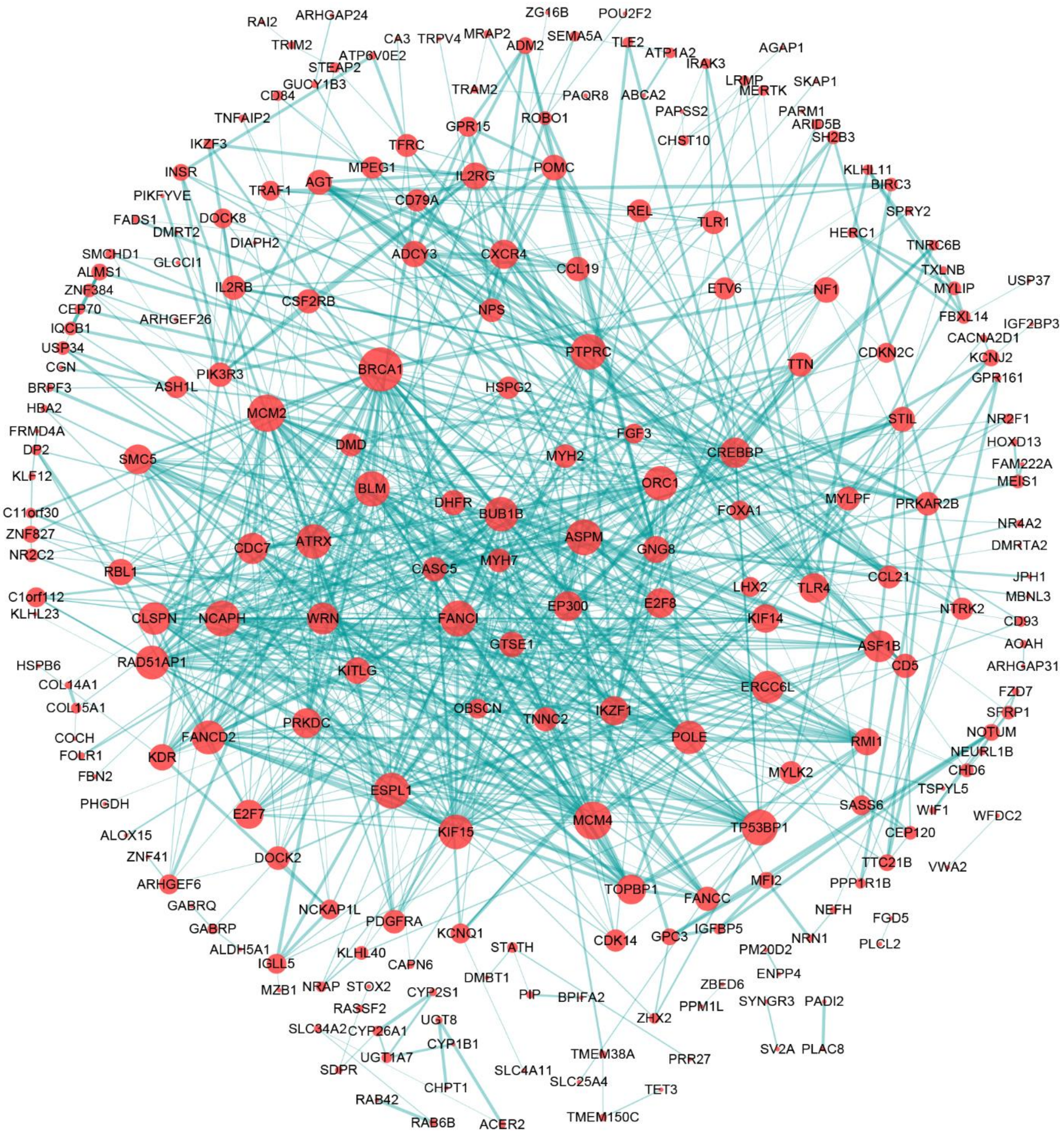

Supplementary Figure 3. The PPI network of low-risk subgroups of up-regulated DEGs genes created by Cytoscape. The node was represented as gene and the edge was deemed as the interactions. The size of the node and edge can be denoted as the degree and combined_score, respectively.
